# Deletion of the *celA* gene in *Aspergillus nidulans* triggers overexpression of secondary metabolite biosynthetic genes

**DOI:** 10.1038/s41598-017-05920-x

**Published:** 2017-07-20

**Authors:** Gea Guerriero, Lucia Silvestrini, Sylvain Legay, Frank Maixner, Michael Sulyok, Jean-Francois Hausman, Joseph Strauss

**Affiliations:** 1 Luxembourg Institute of Science and Technology (LIST), Environmental Research and Innovation (ERIN) Department, Esch/Alzette, L-4362 Luxembourg; 2 University of Natural Resources and Life Sciences Vienna (BOKU), Department of Applied Genetics and Cell Biology, Fungal Genetics and Genomics Unit, BOKU Campus, Tulln/Donau, A-3430 Austria; 3 European Academy of Bozen/Bolzano (EURAC), Institute for Mummies and the Iceman, Bolzano, 39100 Italy; 4 University of Natural Resources and Life Sciences Vienna (BOKU), Department for Agrobiotechnology (IFA-Tulln), A-3430 Tulln, Austria

## Abstract

Although much progress has been made in the study of cell wall biosynthetic genes in the model filamentous fungus *Aspergillus nidulans*, there are still targets awaiting characterization. An example is the gene *celA* (ANIA_08444) encoding a putative mixed linkage glucan synthase. To characterize the role of *celA*, we deleted it in *A. nidulans*, analyzed the phenotype of the mycelium and performed RNA-Seq. The strain shows a very strong phenotype, namely “balloons” along the hyphae and aberrant conidiophores, as well as an altered susceptibility to cell wall drugs. These data suggest a potential role of the gene in cell wall-related processes. The Gene Ontology term Enrichment analysis shows increased expression of secondary metabolite biosynthetic genes (sterigmatocystin in particular) in the deleted strain. Our results show that the deletion of *celA* triggers a strong phenotype reminiscent of cell wall-related aberrations and the upregulation of some secondary metabolite gene clusters in *A. nidulans*.

## Introduction

The cell wall is a structure involved in important stages of fungal growth and morphogenesis. Several studies in the literature have shown how perturbations at the cell wall-level trigger dramatic effects on growth^1^. Despite the importance of fungal cell walls and despite the great advances made in the field, there are still missing pieces in our understanding of cell wall dynamics in filamentous fungi. Some cell wall biosynthetic genes, for example, are still uncharacterized (a detailed inventory of *Aspergillus nidulans* cell wall-related genes has been previously published^2^). The chief polysaccharides in the cell wall of the model organism *A. nidulans* are β-glucans (β-1,3-, β-(1,3;1,4)- and β-1,6-glucans), chitin and α-1,3-glucans. No characterization is yet available for the proposed mixed linkage glucan synthase gene *celA* ANIA_08444^2^, while much is known about the chitin and α-1,3-glucan biosynthetic genes in *A. nidulans* ^3–8^. In *A. nidulans* 6 chitin synthase genes have for instance been identified which belong to classes I to VI^3–7^. *chsC* and *chsA* were proven to play an important role in hyphal wall integrity and conidiophore development^4^, while *chsB* and *chsD* are involved in hyphal growth and conidiation^5^. Interestingly two of the identified chitin synthases possess a myosin-motor head domain^7^, which was shown to be important for the interaction with the cytoskeleton and to play a role for the correct delivery and insertion in the plasma membrane.

Recently, a study on *A. fumigatus* has characterized a protein with significant similarity to ANIA_08444 termed Tft1, an enzyme shown to be responsible for the production of β-(1,3;1,4)-glucans in this organism^9^.

**Figure 1:**
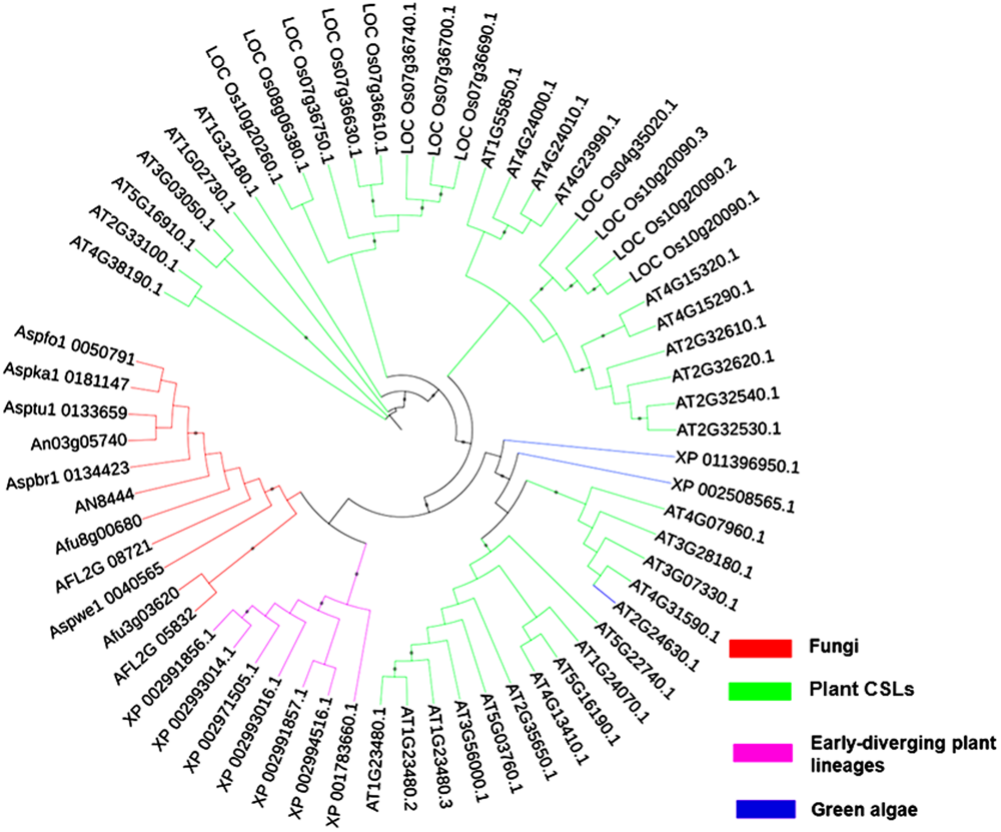
Neighbor-joining tree (bootstraps = 1000) of CelA, its fungal and plant orthologs. Accession numbers are indicated in the tree.

The protein encoded by *celA* is distantly related to plant cellulose synthase-like proteins (CSLs; Fig. 1), it is orthologous to the characterized *A. fumigatus* mixed-linkage glucan synthase Tft1^9^, whose deletion causes no obvious phenotype in *A. fumigatus*, except for a modest increase in virulence.

To characterize the role of *celA* in the growth and development of the model filamentous fungus *A. nidulans*, we here sought to provide transcriptomic data of a strain which lacks the gene *celA* (the strain is hereafter referred to as *celA*∆).

In the present study, we provide experimental evidence for a relationship existing between the deletion of the gene *celA* and the upregulation of genes involved in the production of secondary metabolites (SM). We propose that *celA* partakes in a mechanism regulating SM production in *A. nidulans*.

## Results and Discussion

### Deletion of *celA* causes a strong phenotype in *A. nidulans*

Transformation of the SAA.111 recipient strain with the replacement cassette containing the *argB* selectable marker yielded three independent transformants; however, subsequent growth cycles of the transformants from conidia on selective medium resulted in one single transformant as capable of stable growth under arginine deprivation. This *celA*Δ strain was analyzed and found to have a single and correct *argB* marker insertion replacing *celA* in the genome (Suppl. Figure 1a; for a schematic representation of the *EcoRI* restriction map in the wild-type and *celA*Δ, see Suppl. Figure 1b). The diagnostic PCRs highlighted the expected shift in MW caused by deletion of *celA* and insertion of the *argB* marker (Suppl. Figure 1c and d).

**Figure 2:**
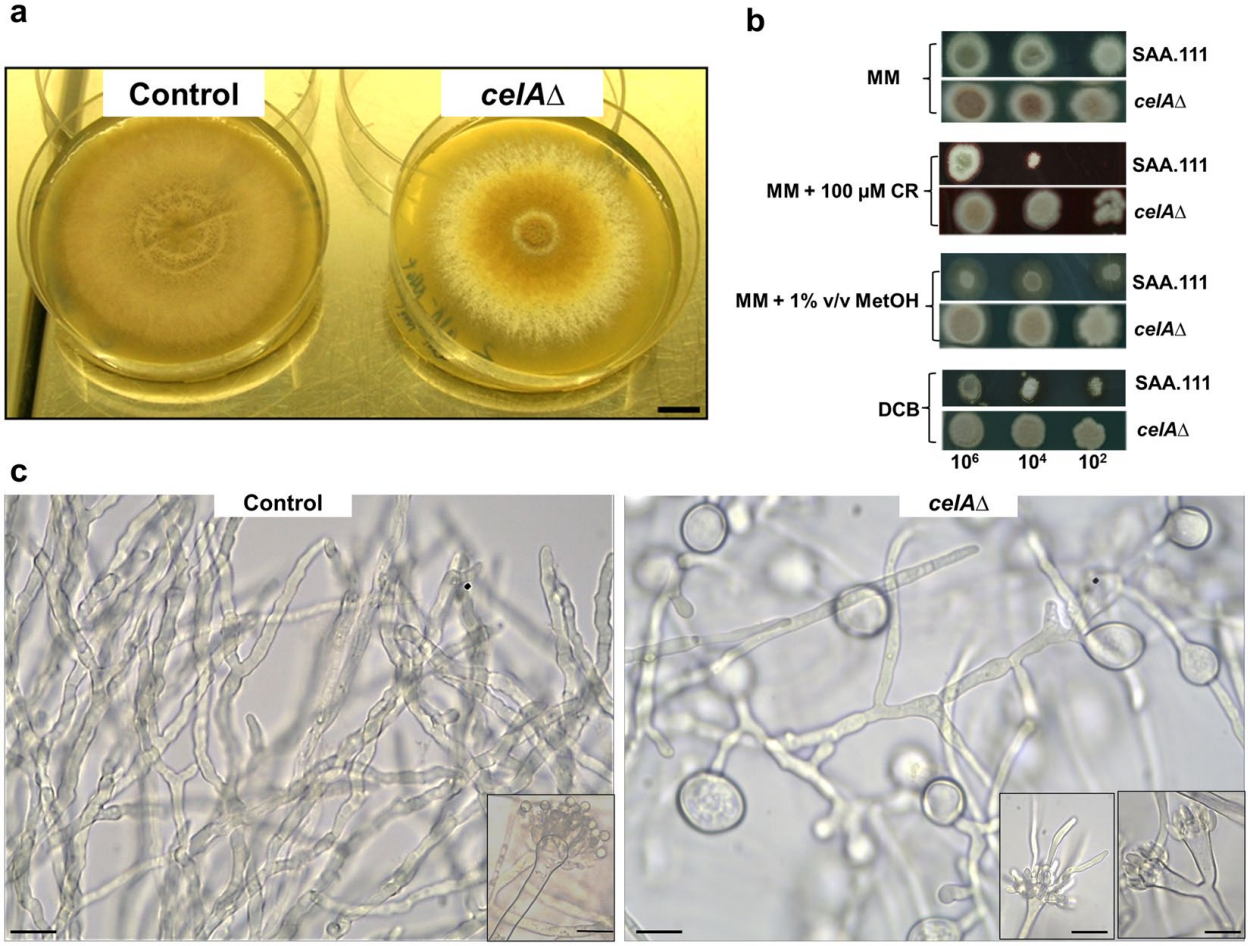
Characterization of the *celA*Δ strain. (**a**) Macrophenotype of the *celA*Δ strain on solid medium. Bar refers to 1 cm. (**b**) Sensitivity to the cell wall drugs CR and DCB. Numbers refer to spore dilutions. (**c**) Balloons along the hyphae and aberrant conidiophores in the *celA*Δ strain. Bars refer to 10 µm.

**Figure 3:**
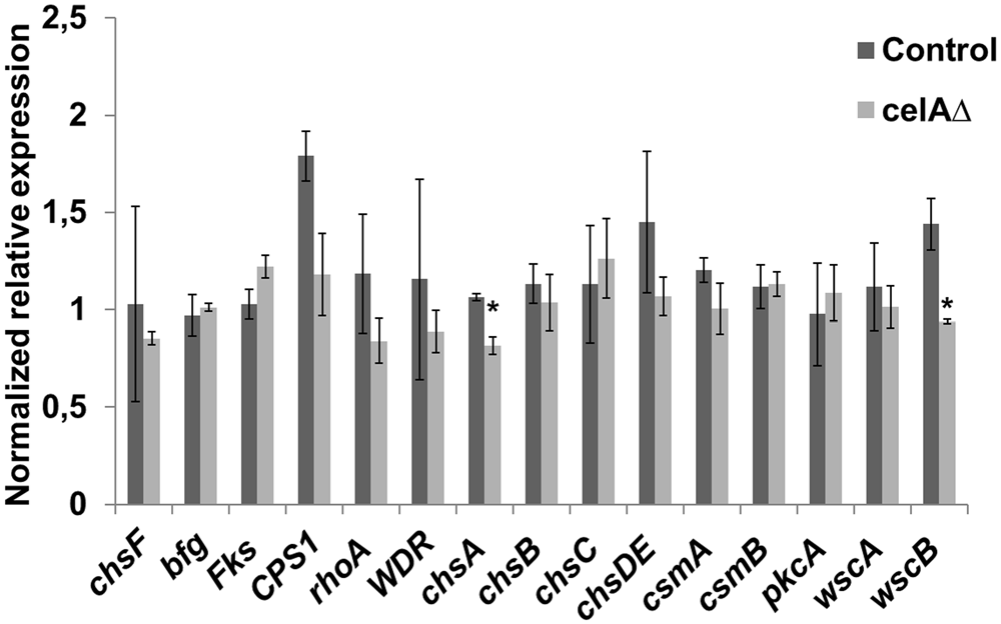
RT-qPCR analysis on a set of cell wall-related genes in the control and *celA*Δ strain. The data were normalized using *rpl3* and *CRP2*. Asterisks indicate statistically significant differences (*p* < 0.05).

When grown on solid medium, the *celA*Δ strain showed a macrophenotype: besides growing more slowly, it developed a colony with an outer sector devoid of pigments, as compared to the control (Fig. 2a). The *celA*Δ strain showed decreased sensitivity to the cell wall drugs Congo Red (CR) and dichlobenil (DCB) (Fig. 2b). Microscopic observations of the hyphae in the *celA*Δ strain revealed frequently swollen regions and “balloons”; at higher magnifications these structures appeared vacuolated (Fig. 2c). Additionally, several conidiophores retrieved from plate cultures were aberrant, since they showed bifurcations and hyphae-like metulae (Fig. 2c insets). It is known that *A. nidulans* strains lacking specific chitin synthase genes of class V and VI also show a “balloon” phenotype^7^, which is indicative of a weakened cell wall. The results here obtained therefore suggest a potential implication of *celA* in cell wall-related processes; however its actual role in cell wall biosynthesis awaits experimental validation. As the deletion of genes triggering phenotypes reminiscent of those observed in *celA*Δ is often accompanied by compensatory alterations in expression of genes involved in cell wall biosynthesis^10^, we tested some of their representatives. As an example, genes involved in chitin and β-1,3-glucan synthesis (Fig. 3) did not show statistically significant changes, with the exception of *chsA* and the cell wall stress sensor *wscB* which were downregulated in *celA*Δ.

### Transcriptome of *celA*Δ

Since a strong phenotype was observed and since the majority of the cell wall biosynthetic genes screened did not show dramatic changes in expression at the RT-qPCR, we decided to perform a transcriptomic analysis to uncover eventual differences in the expression of genes acting in other cellular pathways.

**Figure 4:**
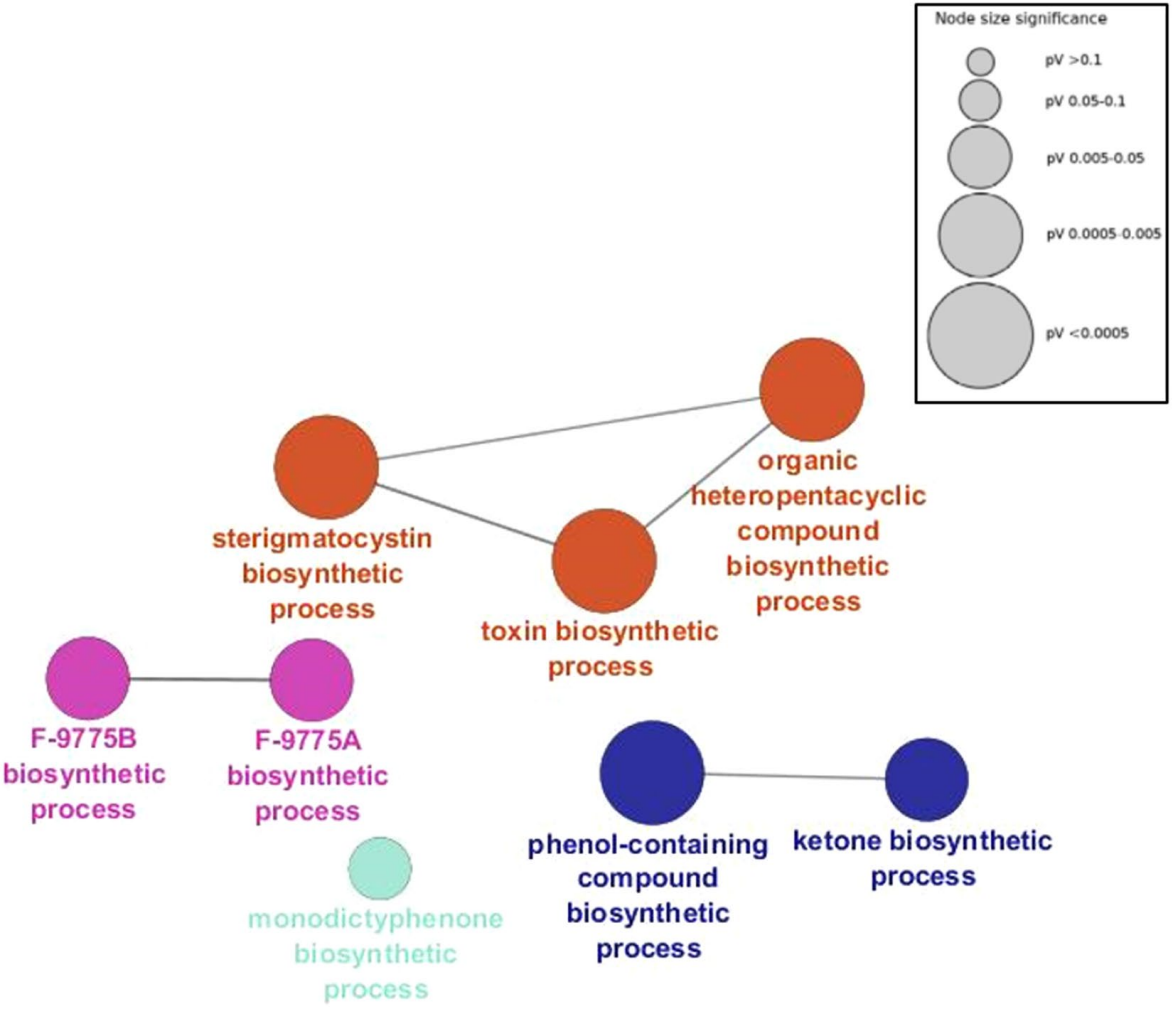
Gene Ontology Enrichment (GOE) analysis of the genes upregulated in *celA*Δ. Gene Ontology (GO) terms sharing similar associated genes are connected with a line. The circle sizes represent different *p*-values (inset).

**Figure 5:**
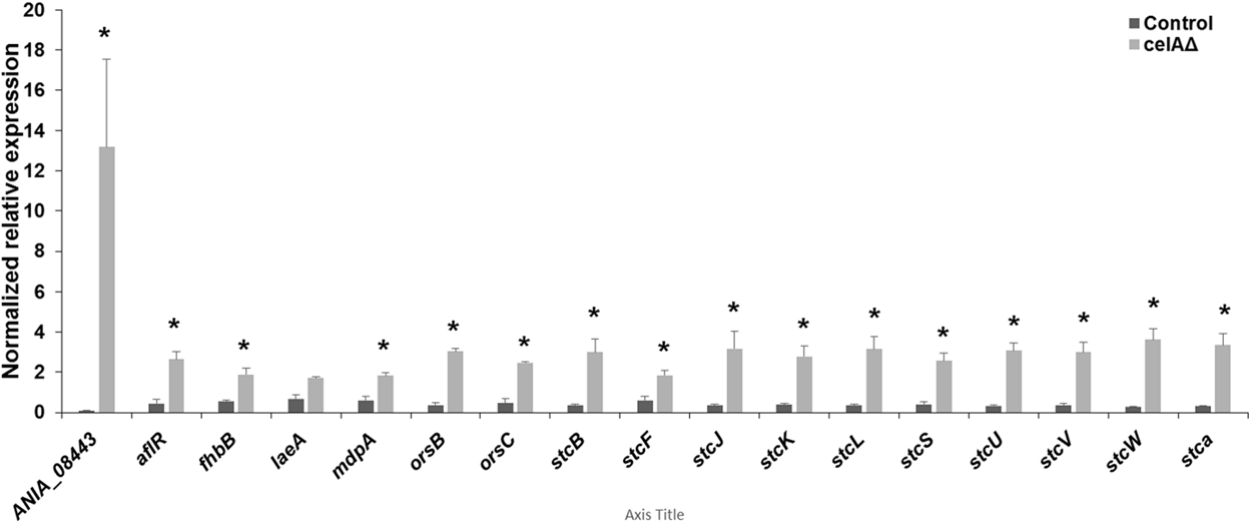
RT-qPCR analysis on ANIA_08443 and a set of secondary metabolism-related genes in the control and *celA*Δ strain. The data were normalized using *rpl3* and *CRP2*. Asterisks indicate statistically significant differences (*p* < 0.05).

After library preparation, quantification and sequencing, we performed data filtering by setting the log_2_ FC > 1 in absolute value (see Materials and Methods). A total of 1305 genes showed statistically significant changes (Suppl. Information). The Gene Ontology term Enrichment (GOE) analysis carried out using ClueGO (in Cytoscape) with the genes upregulated in *celA*Δ (613 in total, see Suppl. Information) revealed an enrichment of functionally-related genes involved in secondary metabolite biosynthesis, such as members of the sterigmatocystin cluster (*stcL*, *stcW*, *stcV*, *stcU*, *stcS*, *stcK*, *stcJ*, *stcF*, *aflR*, *stcB*, *stcA*,), as well as genes involved in yellow-polyketide F9775A/F9775B biosynthesis (*orsA*, *orsB*, *orsC*) and monodictyphenone biosynthesis (*mdpA*, *mdpI*, *mdpH*) (Fig. 4; Suppl. Information). Additionally, two putative SM regulatory genes were also responding to the *celA*Δ deletion by up-regulation (*laeA*, *fhbB*). The results obtained with the GO Term Finder at the AspGD confirmed the enrichment of transcripts involved in secondary metabolic processes in *celA*Δ, with 19 genes involved in toxin biosynthesis (Suppl. Information). The targeted gene expression analysis via RT-qPCR performed on secondary metabolism-related genes (involved in sterigmatocystin, yellow-polyketide F9775A/F9775B and monodictyphenone biosynthesis) confirmed upregulation in *celA*Δ (Fig. 5). Notably, the analysis with the GO Term Finder highlighted a downregulation of genes involved in organonitrogen compound catabolic process: 20 of the identified genes belong to the cellular amino acid catabolic process (Suppl. Information). These data show that, in a manner analogous to what proven in *A. fumigatus* ^11^, an alteration of the cell wall status has an effect on primary metabolism (the primary metabolism is affected with consequent lower abundance of amino acids). Such a phenomenon may be operating in the *celA* mutant, where a stress response is unleashed as a consequence of *celA* deletion and therefore a downregulation of genes belonging to the amino acid biosynthetic process is observed.

**Figure 6:**
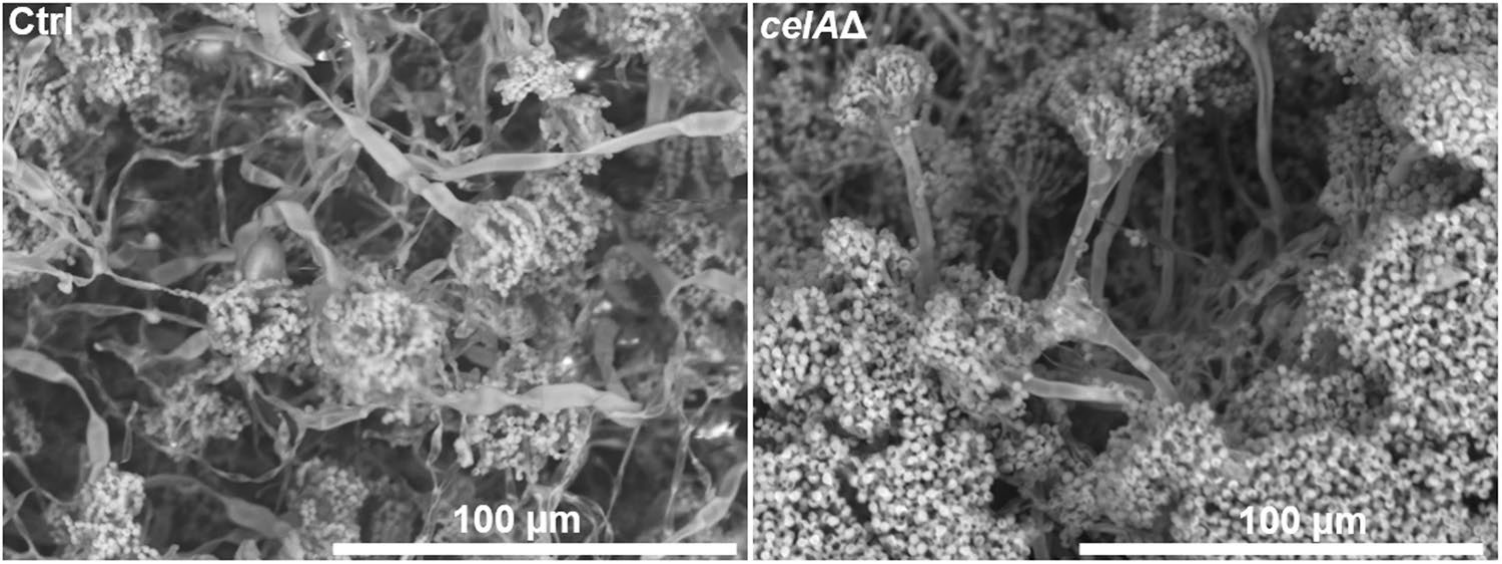
Scanning electron microscope pictures of the control (SAA.111) and *celA*Δ. The deleted strain forms more conidiophores and conidia than the control (the cultures are 4 days old).

**Figure 7:**
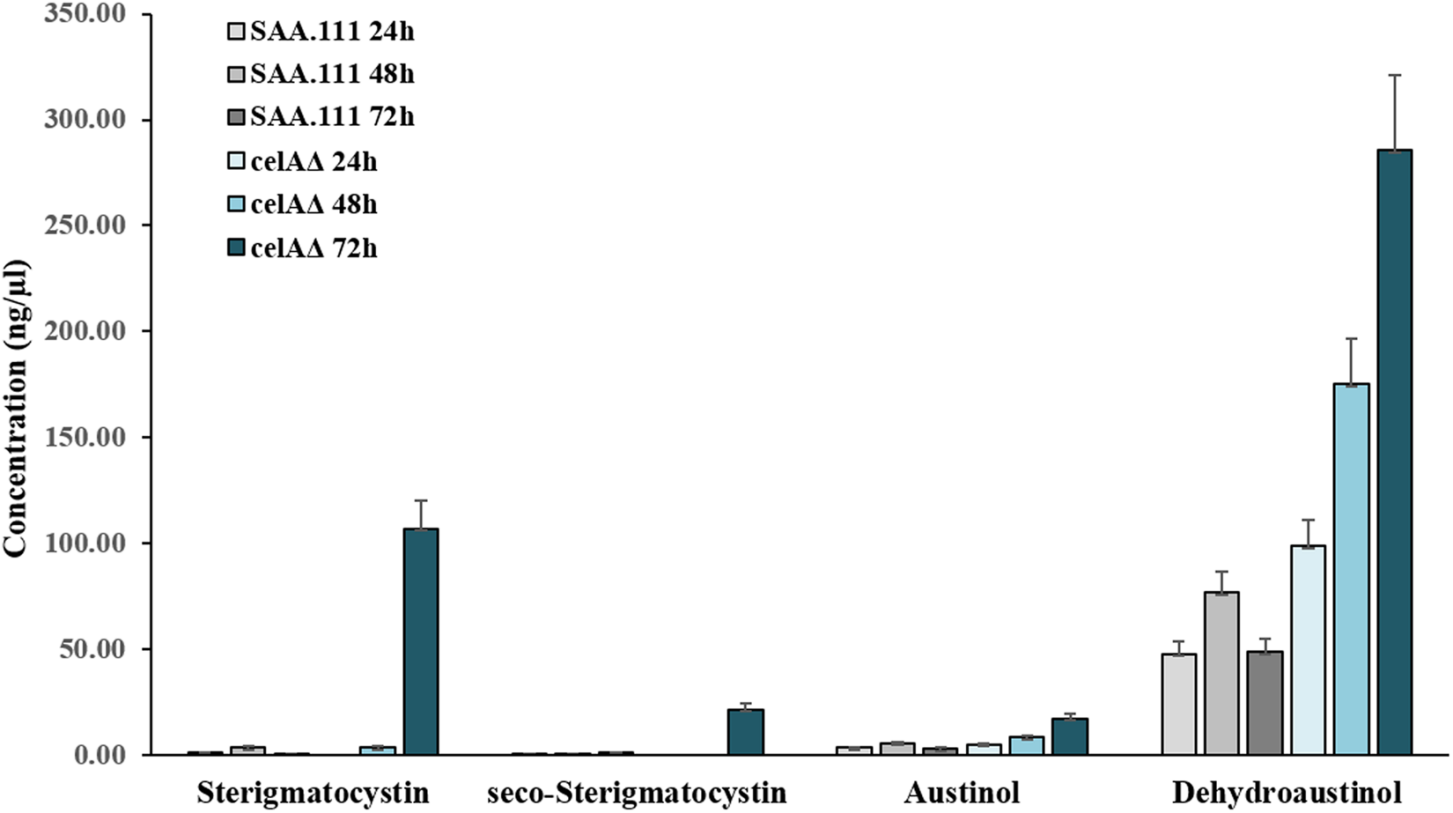
Quantification of some secondary metabolites in the control and *celA*Δ strain.

High-resolution imaging using scanning electron microscopy (SEM) highlighted a higher number of conidia and conidiophores in *celA*Δ (Fig. 6). In this respect, it should be noted that in the RNA-Seq dataset four conidiation-related genes with increased expression in the mutant are present (Suppl. Information). These genes are: ANIA_05086 (log_2_FC*celA*Δ/ctrl = 3.246), ANIA_10628 (log_2_FC*celA*Δ/ctrl = 3.236), ANIA_05015 (log_2_FC*celA*Δ/ctrl = 2.509), *flbD* (log_2_FC*celA*Δ/ctrl = 1.502). Interestingly, *flbD* is a member of the fluffy genes required to activate *brlA* and some fluffy mutants are known to display failure to produce sterigmatocystin^12^. The higher expression of the Myb transcription factor *flbD* may explain the higher conidiation observed in the mutant, however this is probably not related to the higher sterigmatocystin biosynthesis-related genes, since it was reported that *flbD* overexpression did not trigger an increase in *stc* transcripts^13^. Some secondary metabolites were quantified using LC-MS/MS at different time-points (24 h–72 h–96 h) and the analysis showed an increase in sterigmatocystin, seco-strigmatocystin, austinol and dehydroaustinol in *celA*Δ which was the strongest at 96 h (the RNA-Seq time-point) (Fig. 7).

In the light of the observed results, it is here proposed that the increased expression (and production) of secondary metabolites is linked to the sensing of a stress caused or mimicked by the deletion of the gene *celA*.

Under laboratory conditions, many fungal SM gene clusters are cryptic and remain dormant based on a chromatin silencing and inactive signaling pathway^14^. They are instead active when the organism is subjected to specific nutritional conditions and/or environmental constraints^15, 16^ and enable the colonization of specific natural niches. Manipulations of growth composition can also trigger increased SM biosynthesis, as recently shown with ionic liquids in *A. nidulans* ^17^. Therefore, the increased expression in SM biosynthetic genes likely reveals a stress status, probably generated by the altered cell wall in *celA*∆. It should be noted that the deleted strain shows increased resistance to cell wall drugs (Fig. 2b): this is not unexpected, since in the literature it has for instance been shown that, in *A. fumigatus*, deletion of *dvrA*, a transcription factor partaking in cell wall integrity signaling, triggers increased resistance to Nikkomycin Z^18^. The phenotypes observed, as well as the altered cell wall drug sensitivity, support the hypothesis of a role of *celA* in a cell wall-related process.

Interestingly, previous studies in the literature on another model fungus, *Hypocrea jecorina*, have shown that a compromised cell wall has consequences on cellulase production: the mutant *Δtmk2* shows increased cellulase production^19, 20^, a feature most likely triggered to favor the growth of a damaged fungal cell, in a manner analogous to what is observed in the *Δtmk3* mutant under solid-state growth^19^. Cellulases (as well as other secreted enzymes) may help form a protein layer functioning as an osmotic stabilizer for fungal cells with damaged cell walls^19^. It is thus already reported in the literature that alterations in the cell wall influence complex cellular processes, namely the production and secretion of cellulases.

In our dataset, the second gene showing the highest expression in *celA*Δ with respect to the isogenic wild type is ANIA_08443, a gene with no known function. This gene shows a log_2_ FC increase in expression of 8.74 (which means it is expressed ca. 429 times more in *celA*Δ than the control). The targeted RT-qPCR analysis confirms upregulation of ANIA_08443 in *celA*Δ (Fig. 5). It should be noted that this gene is adjacent to the *celA* locus and up-regulation could be a coincidental consequence of marker gene insertion at the place of the *celA* gene. However, the BLASTP analysis indicates that the corresponding protein has a DUF3431 domain and predicted to possess a transmembrane region (prediction made with TMHMM v2.0 http://www.cbs.dtu.dk/services/TMHMM/ and Phobius http://phobius.sbc.su.se/) and to expose a large portion including the C-terminus to the cell’s exterior (Suppl. Information; residues 27–384 according to TMHMM and 37–384 according to Phobius). Therefore, we cannot exclude that this protein may represent a sensor of the cell wall status. More specifically, this uncharacterized protein may represent a potential candidate involved in cell wall status sensing and signal transduction to the cell’s interior. Its role in signal transduction and activation of SM biosynthetic gene clusters awaits validation via functional studies, nevertheless the results shown pave the way to future studies on *celA*, as well as on the link between cell wall alterations and SM production in *A. nidulans*. In this respect we believe important to mention that a DUF3431 protein was shown to be upregulated upon salt stress in the halotolerant fungus *A. glaucus* ^21^ and to confer increased stress tolerance when expressed heterologously in *Arabidopsis* plants.

Future functional studies should provide evidence for an implication of this gene in cell wall status sensing and SM production in *A. nidulans*.

## Materials and Methods

### Deletion of ***celA*** in *A. nidulans*

The strain used in this study is SAA.111 (genotype *veA1; biA1;* Δ*argB:: trpC; riboB2; pyroA4; wA3*)^22^. Gene replacement of ANIA_08444 was performed with the double joint-PCR (DJ-PCR)^23^: the cassette containing the selectable marker *argB* (ANIA_04409) was inserted to replace the entire locus ANIA_08444. The *argB* gene was amplified from plasmid pMS12^24^. Transformation was carried out as described previously^22^.

The primers used for the DJ-PCR are indicated in Suppl. Information.

PCRs to create the replacement cassette were performed with the Phusion High-Fidelity PCR Master Mix, according to the manufacturer’s instructions. Optimal annealing temperatures were computed with the NEB Tm calculator v1.9.4 (http://tmcalculator.neb.com/#!/).

Deletion was checked via Southern blotting, which provided clear evidence of deletion (Suppl. Figure 1a), however RNA-Seq, due to its high sensitivity, detected very low background expression levels of *celA* (average 3.8 RPKM). This expression level was however negligible and probably due to contaminating residual heterokaryons; it was not relevant to the further characterization of *celA*∆, whose phenotype is reminiscent of already characterized cell wall mutants^7^. Southern blotting (DNA cut with *EcoRI* and DIG-labelled probe amplified with primers celA Upstream Fwd and celA Downstream Rev), diagnostic PCR on genomic DNA (gDNA) and RT-qPCR were performed as described previously^25^. Diagnostic PCRs using gDNA were performed with the Q5 Hot-Start High-Fidelity Master Mix, using the primers celA nested Fwd and celA nested Rev (Suppl. Information); the annealing temperature was set at 64 °C with an extension of 3 minutes and a total of 30 amplification cycles on a Veriti Thermal Cycler. The primers of the reference genes *rpl37*, *rpl3*, *CRP2* and actin used for the RT-qPCR have been previously reported^24^. The RT-qPCR primers used to amplify the additional reference gene *H2B*, as well as those relative to the secondary metabolism genes are in Suppl. Information. Optical microscopy and growth test in the presence of cell wall drugs were performed as previously described^26^.

### RT-qPCR, RNA-Seq and bioinformatics

Libraries were prepared from 3 µg of total RNA extracted from the mycelium after 3 days of growth at 37 °C with the SMARTer Stranded RNA-Seq kit (Clontech). The isolation of mRNAs was performed using the Illumina beads and the TruSeq protocol (Illumina), as previously described^27^. The synthesis of cDNA and shearing were performed with ten ng of mRNA, according to the manufacturer’s instruction. The enrichment step was carried out using 14 cycles of PCR. The libraries were then checked using a 2100 Bioanalyzer (DNA High sensitivity Kit) to estimate the average fragment size. Library quantification was performed using the KAPA library quantification kit (KAPA Biosystems) and a ViiA7 Real-Time PCR System (Life technologies). The pooled libraries (at a concentration of 20 pM) were sequenced on an Illumina MiSeq (MiSeq reagent kit V3, 150 cycles) generating 76 base pairs paired-end reads. Raw sequences have been deposited at the NCBI Gene Expression Omnibus (GEO), http://www.ncbi.nlm.nih.gov/geo, under the accession number GSE94110 (link to access the data https://www.ncbi.nlm.nih.gov/geo/query/acc.cgi?token = qnojukmmnzifxqv&acc = GSE94110).

Raw FASTA files were imported in CLC Genomics Workbench 9.0.1. Sequences were filtered and trimmed according to the following criteria: sequence length > 35 base pairs (bp), sequence quality score < 0.01, no ambiguity in the sequence, trimming using Illumina adaptors, hard trim of 8 bp at the 5′ end and 2 bp at the 3′ end, resulting in a longest length of 64 bp. For each library, the mapping against the *A. nidulans* FGSC A4 transcriptome (downloaded at the http://www.aspergillusgenome.org/download/sequence/A_nidulans_FGSC_A4/current/) was performed with the following settings: a maximum hit per reads of 3, similarity fraction = 0.9, a length fraction = 0.9, a mismatch, insertion and deletion cost of 3 (stringent mapping). The expression values were then calculated using the RPKM method^28^. In order to highlight the differentially expressed genes, a t-test with 2 groups (control and *celA*∆, each composed of three biological replicates) was performed. Only genes with a *p-*value below 0.05 were selected. A cut-off threshold was also applied to the fold change (log_2_ FC absolute value > 1).

The GOE analysis was carried out using loci with log_2_ FC > 1 (i.e. overexpressed in *celA*∆) using ClueGO (v2.3.2)^29^ within Cytoscape (v3.4.1) with the following parameters: gene ontology from level 3 to level 8, kappa score set at 0.6, *p*-value < 0.05, enrichment analysis with a Benjamini-Hochberg correction. The GO analysis was also carried out using the GO Term Finder at the AspGD (http://www.aspergillusgenome.org/cgi-bin/GO/goTermFinder).

Full-length protein sequences (accessions indicated in the tree) were aligned with ClustalOmega (http://www.ebi.ac.uk/Tools/msa/clustalo) and the alignment was submitted to PHYML (http://www.phylogeny.fr). The tree was visualized with iTOL-Interactive Tree Of Life (http://itol.embl.de/).

### SEM and metabolite profiling by LC-MS/MS

The analysis at the SEM was carried out as previously described^26^. LC-MS/MS screening of target fungal metabolites was performed with a QTrap 5500 LC-MS/MS System (Applied Biosystems, Foster City, CA) equipped with a TurboIonSpray electrospray ionization (ESI) source and an 1290 Series HPLC System (Agilent, Waldbronn, Germany). Chromatographic separation was performed at 25 °C on a Gemini^®^ C18-column, 150 × 4.6 mm i.d., 5 µm particle size, equipped with a C18 4 × 3 mm i.d. security guard cartridge (all from Phenomenex, Torrance, CA, US). The chromatographic method, as well as the chromatographic and mass spectrometric parameters, have been previously described^30^, but the method has in the meantime been expanded to cover 650 metabolites (manuscript in preparation).

## Electronic supplementary material

Supplementary Figure 1

Supplementary Information
