# Supplementary material for: Deletion of the celA gene in Aspergillus nidulans triggers overexpression of secondary metabolite biosynthetic genes
Source: Sci Rep. 2017 Jul 20;7:5978. doi: 10.1038/s41598-017-05920-x (PMC5519750; doi:10.1038/s41598-017-05920-x)
Supplement: Supplementary file 1 — Supplementary Figure 1 [file 41598_2017_5920_MOESM1_ESM.pdf]

Deletion of the *celA* gene in *Aspergillus nidulans* triggers overexpression of secondary metabolite biosynthetic genes

Gea Guerriero[1,*], Lucia Silvestrini[2], Sylvain Legay[1], Frank Maixner[3], Michael Sulyok[4], Jean-Francois Hausman[1], Joseph Strauss[2,*]

[1]Luxembourg Institute of Science and Technology (LIST), Environmental Research and Innovation (ERIN) Department, Esch/Alzette, L-4362, Luxembourg.
[2]University of Natural Resources and Life Sciences Vienna (BOKU), Department of Applied Genetics and Cell Biology, Fungal Genetics and Genomics Unit, BOKU Campus Tulln/Donau, A-3430, Austria.
[3]European Academy of Bozen/Bolzano (EURAC), Institute for Mummies and the Iceman, Bolzano, 39100, Italy.
[4]University of Natural Resources and Life Sciences Vienna (BOKU), Department for Agrobiotechnology (IFA-Tulln), A-3430 Tulln, Austria.

*****gea.guerriero@list.lu; joseph.strauss@boku.ac.at

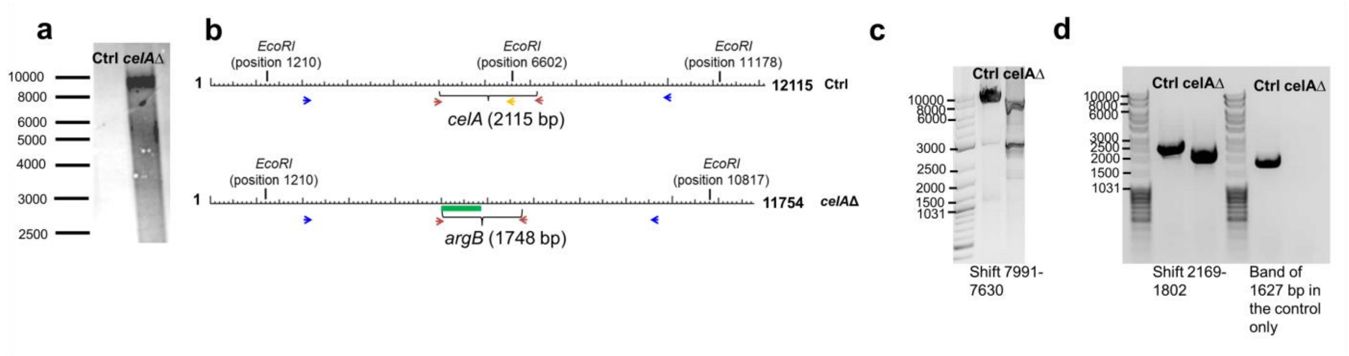

Supplementary Figure 1. Molecular validation of *celA*Δ. a) Southern blotting (band size 9607 bp). b) *EcoRI* restriction map of the *celA* genomic region (+/- 5 kbps) with details of celA nested Fwd and celA nested Rev (in blue), celA diagnostic Fwd and celA diagnostic Rev (in red) and celA specific Rev (in yellow). The probe (815 bp) used for the Southern blotting (amplified with the primer pair argchimera Fwd and arg Southern Rev, Suppl. Information) is indicated in green. c) PCR on the control and *celA*Δ strain using primers celA nested Fwd and celA nested Rev. d) PCRs on the control and *celA*Δ strain using primers celA diagnostic Fwd and celA diagnostic Rev and celA diagnostic Fwd and celA specific Rev.
